# An Integrated NLP‐ML Framework for Property Prediction and Design of Steels

**DOI:** 10.1002/advs.202521457

**Published:** 2026-04-07

**Authors:** Kiran Devraju, Adithya Umanath Rai, Jose Thomson, Marzie Ghorbani, Shujing Zhao, Nick Birbilis

**Affiliations:** ^1^ Faculty of Science, Engineering and Built Environment Deakin University Waurn Ponds VIC Australia; ^2^ College of Systems and Society The Australian National University Acton ACT Australia; ^3^ ARC Research Hub for Australian Steel Innovation Wollongong Australia

**Keywords:** artificial intelligence, machine learning, materials informatics, steel, sustainable design

## Abstract

This paper introduces a data‐driven framework designed to accelerate the property prediction, and hence the discovery and development of steels. The study leverages natural language processing and machine learning techniques to analyse a dataset containing steel compositions, (thermo)mechanical processing, and mechanical properties. By integrating unsupervized machine learning for process classification (via natural language processing‐assisted clustering) and supervised regression models for property prediction, the framework enables an efficient exploration method to study steels. A predictive accuracy of R^2^ > 0.85 was achieved, with mean absolute errors <15 MPa for both yield and ultimate tensile strength. A cloud‐based graphical user interface was developed to facilitate user interaction, allowing researchers to input steel processing and composition to receive predictive insights on mechanical properties. The findings demonstrate the framework to support circular economy principles by reducing trial‐and‐error experimentation, with a view to accelerating the design of steels, and promoting sustainable steel innovation.

## Graphical abstract

This study presents a data‐driven framework that uses language‐processing techniques to interpret steel processing descriptions and machine‐learning models to predict mechanical properties. By organising complex process histories into meaningful groups and enabling rapid property forecasts, the work supports faster, more informed steel design through an accessible, user‐friendly digital tool.

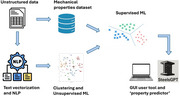

## 1 Introduction

The development of advanced steels with tailored mechanical properties is foundational to innovations in all of energy, transportation, infrastructure, and sustainable materials systems. Yet the traditional cycle of trial‐and‐error alloying, processing, and characterisation remains slow, costly, and susceptible to human bias. In contrast, data‐driven materials design promises to accelerate discovery by mining large, heterogeneous datasets, uncovering hidden structure‐property relationships, and guiding experiments with predictive models. This perspective aligns with foundational materials informatics frameworks, wherein robust descriptors, curated datasets, and machine learning (ML) models enable rapid, data‐centric property prediction and materials understanding [1]. Such approaches can reduce experimental burden, enable exploration of high‐dimensional compositional spaces, facilitate transfer learning across alloy systems, and integrate with physics‐based models—all of which are important on the basis that alloy design inherently includes multicomponent, multiscale complexity [2]. In the utility of data‐driven materials design—specifically alloy design—challenges remain. Such challenges include: dataset quality, interpretability of models employed, and the complexity of classifying alloy processing/process histories [3].

A particular challenge in the case of data‐driven materials design for steels is how to ‘make sense’ of what are typically rather complicated datasets. The study herein explores a curated dataset with 3234 entries. Whilst such a dataset is by no means capturing the complete amount of data published in the field, it is considered a large dataset (noting that a recent study related to predicting the properties of steels utilized a dataset of 312 entries [4], and another utilized 245 entries [5]). Each alloy entry has a corresponding (thermo) mechanical processing condition, which is described by text (and not numerically coded); for example, “13 mm round bar oil quenched 830°C, and tempered at 540°C”. The myriads of processing conditions for steels include many different quenching media, temperatures from which steels are quenched, tempering conditions, often complex (thermo)mechanical processing, and the possibility of additional processes such as carburizing. The number of variables at play, and how they may interact with alloy composition, makes a human‐level interpretation of such large datasets an impossibility. Additionally, the descriptive nature of processing conditions, i.e., such descriptions being text inputs, also frustrates the ability to utilize data science tools to ‘make sense’ of large databases. In this context, the utility of natural language processing (NLP) to interpret large volumes of text descriptions has been identified as critical [6]. Recent advances in language model‐based representation learning further support the use of semantic embeddings for materials‐related text, demonstrating that transformer‐derived descriptors can capture domain‐specific processing and structural information [7]. For example, if NLP tools—specifically the process of so‐called unsupervised machine learning – can be harnessed to interpret text descriptions of steel processing conditions, then this will allow a means to classify (and ‘cluster’) the steel processing conditions. Such an approach was recently demonstrated by Bhat et al., [8] in the comparatively simple case of aluminium alloys, along with Ghorbani et al., [9] for the case of magnesium alloys. The application of NLP approaches to interpret the processing conditions of broad and diverse types of steels is an essential step to subsequent approaches for predicting the mechanical properties of steels.

The application of supervised machine learning as a means to predict the properties of materials, specifically materials not previously explored empirically, has recently become widespread [1]. The approach includes training models via supervised machine learning methods (i.e., regressors, neural networks, ensemble models, etc.) in order to link processing + compositional descriptors → mechanical properties [10]. There are numerous examples of such work, for example, regarding the property prediction of aluminium alloys [11, 12, 13], magnesium alloys [14, 15], and high entropy alloys / multi principal element alloys [16, 17] (where predictions of ultimate tensile strength, yield strength and ductility were possible). Rather than introduce all of the methods involved in the prediction of mechanical properties of alloys via supervised ML, readers are best served by exploring the detailed methodologies such as those in [14].

Prior applications of NLP and unsupervised learning in metallic materials has largely focused on comparatively uniform alloy families—such as aluminium and magnesium—where processing descriptions are less heterogeneous, and the complexity of thermomechanical histories is considerably lower [8, 9]. Foundational work in materials informatics has demonstrated that carefully engineered descriptors, high‐quality datasets, and reproducible validation practices are central to effective data‐driven materials discovery [1, 7]. At the same time, advances in representation learning and neural architectures—including graph‐based models and embedding‐based approaches ‐highlight the expanding role of learned, domain‐aware representations in capturing complex relationships within unstructured or heterogeneous materials information [18, 19]. Motivated by these developments, the present work applies semantic text embeddings and hierarchical clustering to more than 3000 diverse steel processing descriptions, generating data‐driven process classes that provide structured, interpretable inputs for subsequent supervised prediction of mechanical properties.

In the case of supervised ML as applied to steels specifically, clustering approaches have been used to classify microstructural and inclusion behaviors [20], and integrated industrial case studies for process‐product prediction in steelmaking [21]; the latter study highlighting the role and potential of ML in the steel industry more broadly. More recently, Kateb et al., explored machine learning‐driven predictive modelling of mechanical properties in diverse steels [4] – where they utilized a database of strength and ductility for 312 steels (which was extracted from the Citrine database). In another study, Shaheen et al., applied ML to predict the mechanical properties of high‐strength steel at elevated temperatures [22]. However, to date, there is a scarcity of efforts to systematically unify process clustering, composition‐property regression, and user‐facing predictive (or design) tools in a single framework. Thus, a gap remains between predictive modelling and deployable alloy analysis and design platforms. There is also a gap in the accessibility of appropriately sized databases of steel properties.

A key contribution of the present study is the presentation of an open‐access database for steels (including processing condition, composition, and mechanical properties) – providing significant utility to the field. In this work, we combine NLP‐derived processing clusters with multimodel supervised learning and quantitative validation on the largest publicly accessible steelproperty dataset available. By benchmarking alternative embeddings, assessing clustering quality through silhouette scoring, and evaluating supervised models via crossvalidation on a substantial heldout test set, the framework provides a rigorously validated and reproducible approach. Together with the open database and user‐accessible interface, this study delivers both methodological robustness and a distinct advance in integrating processing, composition, and mechanical property prediction for steels. Finally, as part of the study, we introduce a user tool that has been termed “SteelsGPT”. The tool is named “SteelsGPT”, not because it functions as a generative large language model, but because it is inspired by the transformative power of such tools to find structure within complex, unstructured data. The workflow presented in this study overall, was possible to wrap into a cloud‐based graphical user interface (GUI), enabling non‐specialists to input process parameters and receive property forecasts – essentially instantaneously. By compressing complex alloy‐process space into interpretable clusters, forecasting key metrics, and democratising access, this study seeks to close a loop between data science and metallurgy for steels—moving toward actionable alloy design. While it is acknowledged that the study is a data‐driven approach (focused on clustering, regression, and model usability), it is noted that future versions may incorporate physics‐based features such as grain size, phase fractions, precipitate morphology, or texture descriptors.

## 2 Methods

### 2.1 Input Data

The data utilized in the present study was curated from both MatWeb [23] and Ansys Granta EduPack (previously CES EduPack) [24]. The data has been compiled into a database that is available at [25].

**FIGURE 1:**
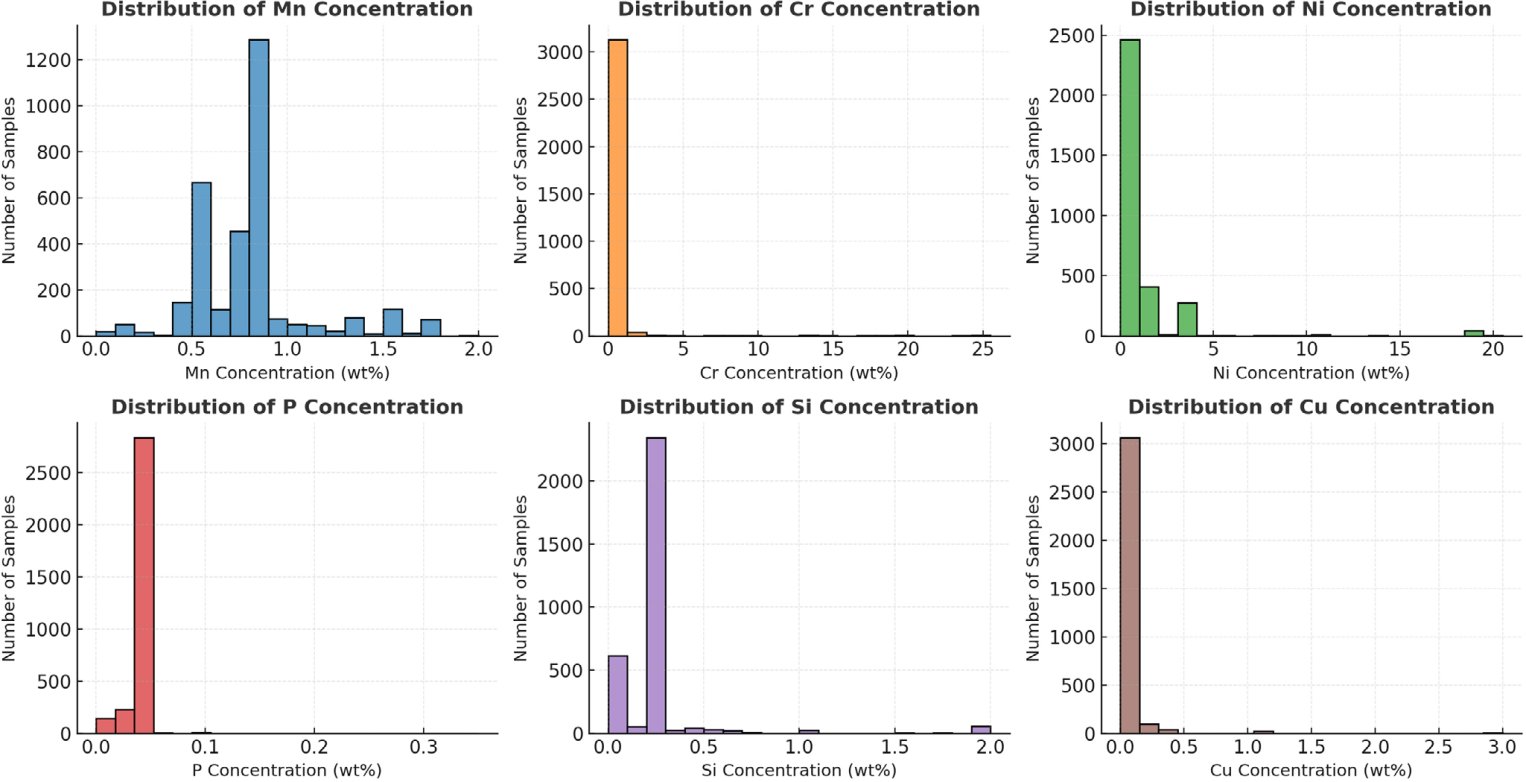
Distribution (as a frequency) for the compositional range of key elements present in steels within the database (including Cu, Cr, Ni, P, Si and Cu).

The database includes steels that contain the following elements, Fe, C, Al, Cu, Mn, N, Ni, Ti, S, Zr, P, Si, V, Mo, Co, Nb, B, Cr. Overall, there are 3234 entries in the database, including steels that represented 346 distinct chemical compositions—highlighting that the processing conditions for steels are both critical and variable (noting there are many alloys of similar or identical compositions in the database, with vastly different properties based on different processing conditions). The spread of output data in the database is provided in Table 1, and the distribution of some of the alloying elements is presented in Figure 1.

**TABLE 1:** Minimum and maximum values of mechanical properties for the steels in the dataset. The data includes a wide range for each property, reflecting the diverse strength–ductility balance across different alloy compositions and processing routes.

Property	Min.	Max.
Ultimate Tensile Strength (MPa)	275	2451
Yield Strength (MPa)	159	2395
Ductility (%)	3	46

### 2.2 Unsupervised Machine Learning

The process of unsupervised ML in this study is presented by means of the tabulated approach in Table 2. The unsupervised ML workflow includes the natural language processing component of the work, applied to rationalize and subsequently cluster the alloy processing conditions that are comprised of diverse text entries.

**TABLE 2:** Workflow adopted in this study for unsupervised machine learning.

Step	Process	Method/Model	Purpose
1	Text Cleaning	Basic string preprocessing	To remove artifacts in any text characters
2	Text Embedding	TF‐IDF	Turn free‐text into numerical feature vectors. Known as natural language processing.
3	Initial Clustering (iterative process)	KMeans DBSCAN HDBSCAN BIRCH	Different methods were explored for their robustness and performance in being able to group similar processing conditions based on text descriptors.
4	Dimensionality Reduction	Truncated SVD (Latent Semantic Analysis)	This is required to project high‐dimensional vectors into 2D for visualisation.
5	Visualisation	Human step: Manual inspection of 2D visualisation	Intuitive human assessment of cluster boundaries and uniqueness, including visual clarity
6	Manual Cluster Merging	Human step: Expert‐driven analysis via a metallurgist	To assess the combination of overlapping/similar clusters, and the metallurgical relevance of the cluster.
7	Final Clustering	The final model selected was the “MiniLM + BIRCH+ Agglomerative” model	Selected based on optimal silhouette score, and interpretable categories ready for supervised ML.

Presenting the process for unsupervised ML in this manner permits the procedures employed to be replicated, and also reveals the steps utilised. The workflow employed readily available computational tools that can be accessed and implemented via Python, and also accessible via large language models (LLMs), including ChatGPT and Microsoft Copilot. Key points regarding the workflow that are emphasized in Table 2 include: the need to iteratively explore the clustering models for suitability, the need for human (expert) level assessment for cluster suitability, and optimisation of clustering using a numerical assessment (in this case, the silhouette score). The final model selected in this work was the “MiniLM + BIRCH+ Agglomerative”, which provided a silhouette score of 0.496 (the highest of all the interpretable clustering process assessed).

The “MiniLM + Birch + Agglomerative” pipeline serves as the unsupervised foundation of the SteelsGPT encoder. SentenceTransformer's all‐MiniLM‐L6‐v2 model (developed by HuggingFace [26]) converts text process descriptions into 384‐dimensional semantic embeddings, capturing contextual meaning between alloy processing routes [27]. These normalised vectors are first grouped utilizing the BIRCH algorithm [28], which efficiently generates micro‐clusters using a hierarchical tree structure that scales to thousands of samples. The resulting centroids are then refined via Agglomerative Clustering to obtain interpretable, semantically coherent macro‐clusters. This hybrid approach balances scalability and contextual accuracy, outperforming purely distance or density‐based approaches for unstructured metallurgical text. This combination significantly enhanced cluster interpretability by capturing both semantic similarity and metallurgical relevance. The MiniLM embeddings effectively represented complex process descriptions in dense vector space, while BIRCH provided efficient preliminary grouping. The agglomerative refinement then merged contextually aligned clusters, revealing distinct processing routes and manufacturing patterns. This layered approach reduced noise and improved stability across iterations, producing clusters that reflected real‐world metallurgical logic. Such hybrid hierarchical strategies have been increasingly adopted in materials informatics and NLP research for improving both computational scalability and domain‐specific understanding [29, 30].

### 2.3 Supervised Machine Learning

In this study, numerous supervised ML models were systematically explored to evaluate their comparative performance in predicting material properties based on clustered process‐composition descriptions. The rationale for exploring the suitability of ML models algorithms stems from the inherent variability and nonlinearity of metallurgical data, where it is difficult to determine a priori if a single model can uniformly outperform others across all target variables. Linear regression and ElasticNet [31] were first employed to establish a baseline, offering interpretability through feature weights and regularization. Subsequently, non‐linear models including as Random Forest (RF), Support Vector Regression (SVR), Multi‐Layer Perceptron (MLP) and Extreme Gradient Boosting (XGBoost) were tested to capture complex, multivariate dependencies between process parameters and resulting mechanical responses. Each model underwent hyperparameter tuning and cross‐validation to permit a robust generalisation and to minimize overfitting. Examples of the use of such models in previous works can be ascertained from recent studies [32, 33, 34, 35, 36].

This exploratory approach was carried out to allow the identification of a supervised ML models architecture balancing predictive accuracy, interpretability, and computational efficiency. Random Forest and MLP demonstrated superior performance for non‐linear behaviour, while ElasticNet maintained value in revealing parameter significance. Such comparative benchmarking aligns with contemporary practices in materials informatics, where multiple supervised models are leveraged to validate predictive robustness and highlight data‐driven insights. This methodological pluralism enables improved understanding of alloy‐property relationships and aids in developing transferable models for alloy design and optimisation [7, 37].

The supervised ML phase of SteelsGPT seeks to predict mechanical properties including as yield strength (YS), ultimate tensile strength (UTS) and ductility—using the processed dataset of 3234 alloys (each represented by 105 numerical and categorical features derived from both composition and cluster embeddings). To help assure robustness and prevent data leakage, the dataset was split into training, validation, and testing subsets using a 70:20:10 ratio, which permits a fair evaluation and generalisability of the predictive models.

The ML models implemented were ElasticNET, Support Vector Regression (SVR), Multi‐Layer Perceptron (MLP), Random Forest (RF), and Extreme Gradient Boosting (XGBoost) – in order to benchmark performance across linear, kernel‐based, neural, and ensemble learning models. This comparative approach allowed evaluation of how different algorithmic assumptions influence prediction accuracy, model stability, and interpretability. It was determined that ensemble models such as RF and XGBoost were particularly well‐suited for the task at hand, due to their ability to handle input data that is non‐linear, in addition to feature interaction and noise, which are common in metallurgical datasets.

The final ML model selection was based on cross‐validated R^2^ and Mean Absolute Error (MAE) metrics, where R^2^ >0.85 and MAE < 15 MPa was deemed suitable as a model performance target. Among the tested algorithms, the Random Forest model showed the most consistent predictive accuracy while maintaining interpretability and computational efficiency – as demonstrated in the Results section.

### 2.4 User Tool

The study herein sought to provide broad access through the development of an interactive web interface – which allows practitioners at any level to use and comprehend the output of the ML models developed herein. The interactive web interface (termed a GUI, graphical user interface) was developed by transforming Python scripts into a straightforward web interface by using Streamlit to construct this utility. The workflow, including Streamlit was selected owing to its integration with the packages Scikit‐learn, pandas, and matplotlib – enabling Streamlit to produce a real‐time presentation of clustering results and property predictions. The access to the Streamlit web interface is readily possible via a web browser [38].

### 2.5 Statistical Analysis

All statistical procedures employed in this study are referred to in the corresponding sections of the study; here they are summarized for completeness.

#### 2.5.1 Pre‐Processing

Compositional and mechanical‐property data were screened for missing entries and anomalous values. Numerical features were normalized or standardized where required for individual machine‐learning models. Text‐based processing descriptions were converted into numerical embeddings before clustering.

#### 2.5.2 Data Presentation

Model performance is reported using standard regression metrics (R^2^, MAE) and visualized through parity and residual plots. As this work is computational and not experimental, no mean ± SD measurements or replicate‐based comparisons were applicable.

#### 2.5.3 Sample Size (n)

The curated dataset contained 3234 steel entries. Unsupervised clustering used all entries (n  =  3234). Supervised ML used a 70:20:10 split into training, validation, and test sets (n ≈ 323 in the test set).

#### 2.5.4 Statistical Methods

Supervised ML models were evaluated using cross‐validation on the training set, followed by final evaluation on the held‐out test set. No hypothesis‐testing or group‐difference statistical tests (e.g., *p*‐values, post‐hoc analyses) were applicable given that this is a predictive modelling study rather than an experimental comparison of independent sample groups.

#### 2.5.5 Software Used

All analyses were performed in Python using standard scientific libraries, including scikit‐learn, pandas, numpy, matplotlib, and sentence‐transformers.

## 3 Results

### 3.1 Clustering of Alloy Processing Conditions

**FIGURE 2:**
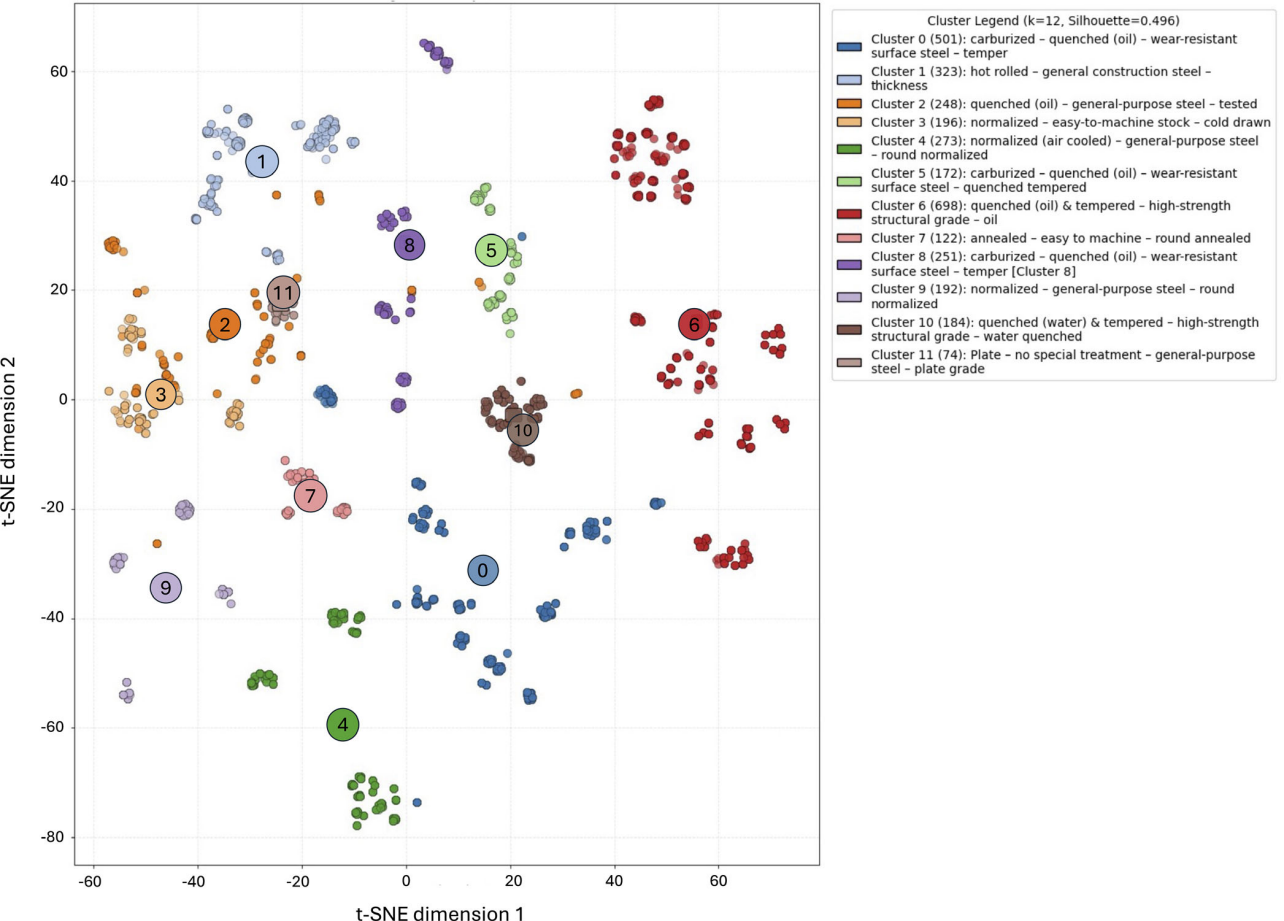
t‐SNE plot of clustered processing conditions according to the implementation of the MiniLM + BIRCH+ Agglomerative model.

A 2D representation of the clustering of processing conditions for steels is presented in Figure 2. The representation is in the form of a t‐SNE (t‐distributed stochastic neighbour embedding) plot, which provides the ability to visually inspect unique clusters, their separation, and uniqueness.

To evaluate the stability of the clustering shown in Figure 2, the MiniLM + BIRCH + Agglomerative workflow was repeated 50 times with different random seeds, yielding highly consistent results (silhouette SD  =  0.009; 92.4% identical assignments). MiniLM embeddings also outperformed TF‐IDF and BERT in silhouette score and cluster cohesion. While expert metallurgical oversight remains essential for human‐expert level assurance that mapping of clusters relates to meaningful processing categories—hence the descriptive synthesis in Table 3—the clustering structure itself is quantitatively robust and only insensitive to initialization or embedding choice.

**TABLE 3:** A description of each cluster observed in Figure 2, with a description of the cluster, along with an interpretable summary of the process condition related to each cluster, and the number of entries from the database (i.e., alloys) in each cluster. Clusters are sorted by their population size.

Cluster	Cluster description	Descriptive process summary (with Typical Temps)	Entries
6	Quenched (oil) & tempered – high‐strength structural steel	**Quenched and tempered**; austenitized at ∼900°C, quenched (oil), then tempered at 200°C–600°C → high strength, moderate ductility.	698
0	Carburized – quenched (oil) – wear‐resistant surface steel	**Case‐hardened (carburized + quenched)**; 900°C–950°C, oil‐quenched surface martensite → hard exterior, ductile core.	501
1	Hot rolled – general construction steel	**Hot rolled**; deformation at 900°C–1100°C, air cooled → medium strength, ferritic–pearlitic.	323
4	Normalized (air‐cooled) – general‐purpose steel	**Normalized**; austenitized ∼900°C, air cooled → fine pearlite/ferrite, balanced strength and ductility.	273
8	Carburized – quenched (oil) – wear‐resistant surface steel (variant)	**Case‐hardened variant**; high‐temperature surface hardening with oil quench → wear‐resistant, strong core.	251
2	Quenched (oil) – general‐purpose steel (tested)	**Quenched**; austenitized ∼900°C, oil quench → martensitic structure, high yield strength.	248
3	Normalized – easy‐to‐machine stock (cold drawn)	**Normalized + cold‐worked**; 850°C–950°C followed by drawing → moderate strength, improved machinability.	196
9	Normalized – general‐purpose steel (round normalized stock)	**Normalized**; medium‐temperature air cool → fine structure, good toughness.	192
10	Quenched (water) & tempered – high‐strength structural steel	**Water‐quenched + tempered**; austenitized ∼900°C, water quenched, tempered 200°C–600°C → very high strength.	184
5	Carburized – quenched (oil) – wear‐resistant surface steel (variant)	**Case‐hardened (carburized + oil quench)**; 900°C–950°C → high surface hardness.	172
7	Annealed – easy‐to‐machine – round annealed stock	**Annealed**; 700°C–900°C, slow furnace cool → soft, ductile, machinable.	122
11	Plate – no special treatment – general‐purpose steel	**As‐rolled / untreated**; baseline ferritic–pearlitic microstructure.	74

As noted in the methodology section, the clustering observed in Figure 2 represents the most ‘optimized’ of the clustering algorithms explored. The “MiniLM + BIRCH+ Agglomerative”, model provided a silhouette score of 0.496. This score is considered robust and highly significant, given the challenge of processing complex, unstructured, free‐text metallurgical descriptions (such as “13 mm round bar oil quenched 830°C, and tempered at 540°C”) where semantic overlap and variability prevent the attainment of a perfect separation score of 1. Crucially, this score represents the highest performance achieved among all configurations deemed metallurgically interpretable, validating our hybrid approach (NLP embedding + scalable clustering + expert knowledge) as essential for successfully structuring this complex, real‐world data. What is observed from Figure 2 are discrete clusters that correspond to unique (and distinct) alloy processing categories; however, to provide metallurgical insights into the nature of the clusters, a synthesis of cluster labels and interpretable process summaries has been compiled in Table 3.

There is a plethora of information that has been truncated into Table 3. This includes an interpretable description of the main classes of processing categories (i.e., clusters) and the relative prevalence of entries in each cluster. It is again noted that to select an unsupervised ML model and process that was faithful to the dataset, human (expert) intervention from the authors was required, along with iterative exploration of unsupervised ML model performance to yield an interpretable list of unique cluster and silhouette score. To the best of the authors' knowledge, a synthesis such as that in Table 3 and Figure 2 has not been presented for steel processing conditions previously. Therefore, a deeper exploration (and presentation) of trends in the database that emerge from the clustering process are elaborated.

**FIGURE 3:**
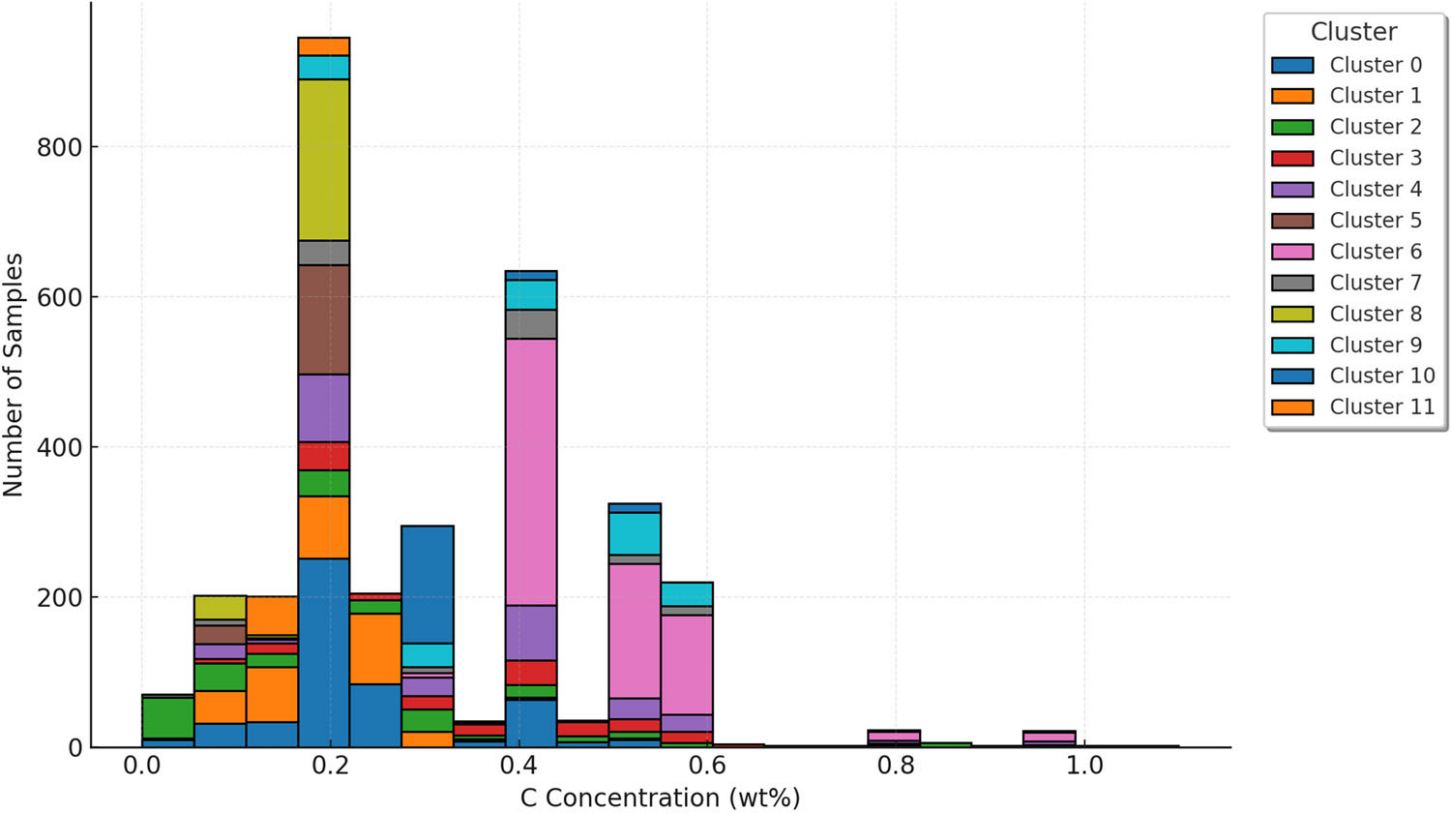
Carbon concentration by cluster, presented as a stacked distribution.

In Figure 3, as one example of working with a database that is now appended with cluster numbers and groupings of processing conditions, the distribution of alloys as a function of carbon (C) concentration is plotted. Each of the distribution bins in Figure 3 is appended with the relative number of alloys from a given cluster. There are several trends that are immediately visible from inspection, such as: Clusters 6 (pink), 7 (gray), and 10 (kingfisher blue) are associated with higher concentrations of C (i.e., C >0.4 wt. %). Conversely, Clusters 0 (blue) and 1 (orange) tended to be associated with lower concentrations of C (i.e., C <0.4 wt. %). Meanwhile, Clusters 2 (green) and 3 (red) were present across the breadth of C concentrations.

**FIGURE 4:**
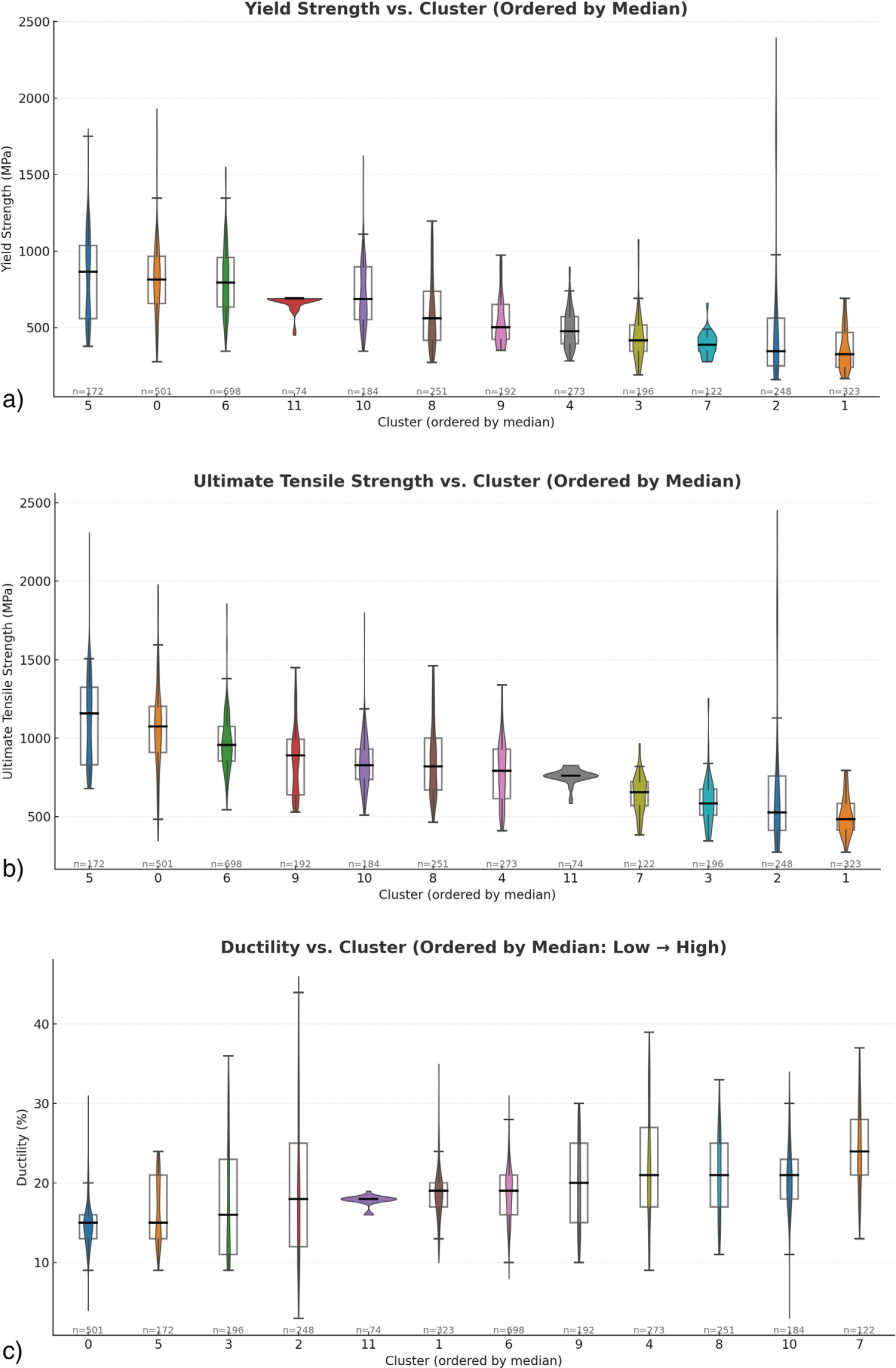
Mechanical properties (a) yield strength, (b) ultimate tensile strength, and (c) ductility, as a function of cluster number ordered by median cluster value.

When plotting the mechanical properties of steels as a function of processing cluster (as seen in Figure 4), there are a number of general trends. At the highest level, some interpretations include the following observations. In the case of yield strength, several clusters dominate high‐strength regions – linked to an association with either a higher Cr, Mo or C content, along with a quenched / tempered process. For (ultimate) tensile strength, the trends are similar to yield strength but with a slightly broader spread, which shows the consistency or variability across clusters. In the case of ductility, the relationships observed are an inversion of the strength trends (which is a particularly clear trend) – whereby clusters that maximize strength typically show lower ductility, capturing the classical strength‐ductility trade‐off, visually.

### 3.2 Machine Learning of Alloy Properties

**FIGURE 5:**
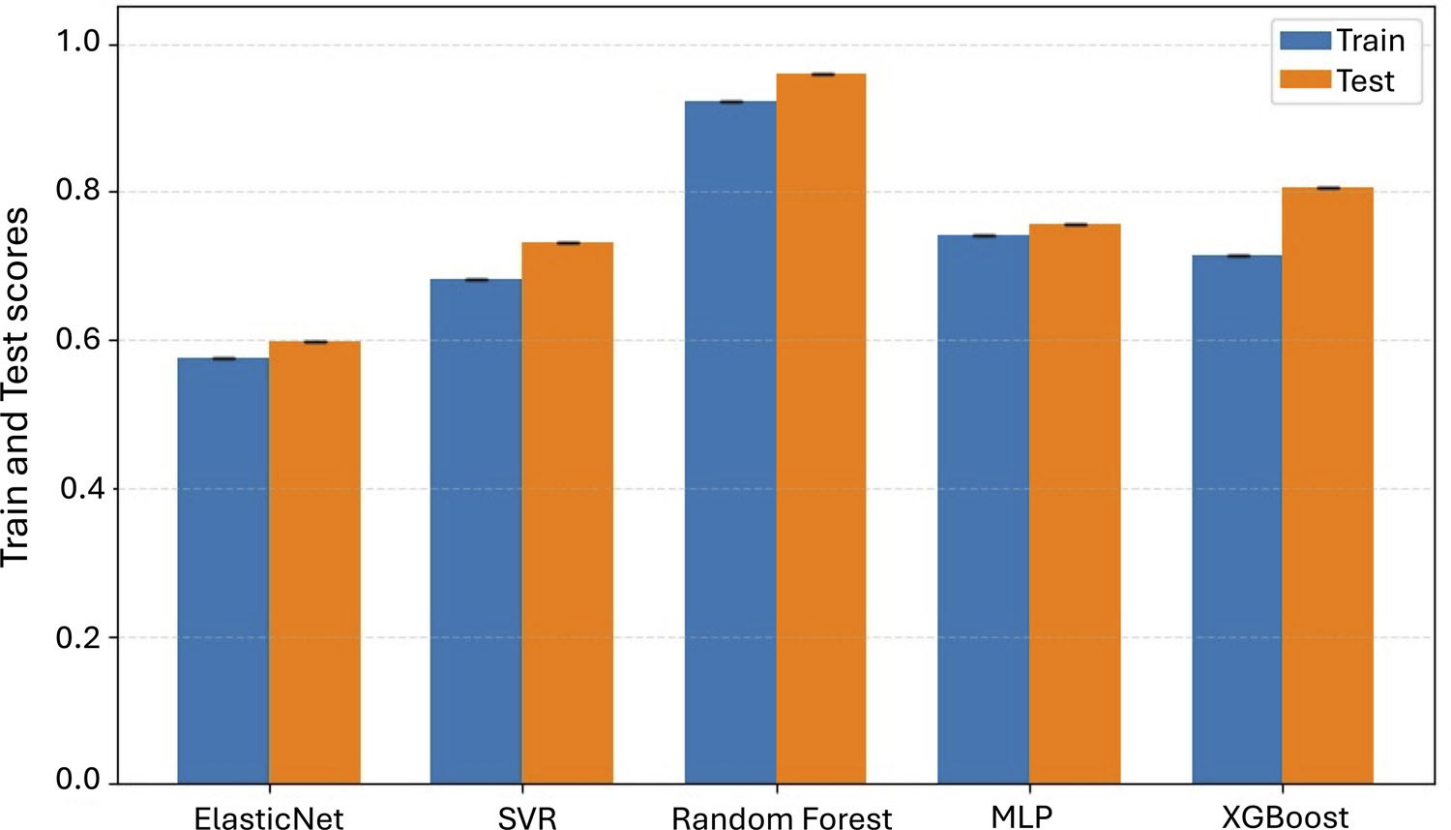
Train and test scores for the various supervised ML models explored in the present study in the prediction of UTS.

As noted in the methods section, the supervised ML to predict alloy properties was carried out by exploring the performance of five models, namely ElasticNet, Support Vector Regression (SVR), Random Forest (RF), Multi‐Layer Perceptron (MLP) and Extreme Gradient Boosting (XGBoost). The relative performance of these models is presented in Figure 5 and Table 4, where it is noted that the training, validation, and testing data splits were carried out using a 70:20:10 ratio. It is noted that the inputs for the supervised ML models include the alloy composition and the associated alloy cluster (whereby the preceding work to generate alloy processing clusters using unsupervised ML was a necessary precursor to supervised ML). The model outputs are ultimate tensile strength, yield strength, and ductility.

**TABLE 4:** A comparison of R^2^ and MAE values for all the supervised ML models explored herein, as applied to the prediction of UTS.

ML model	Overall R^2^ (Val)	Overall R^2^ (Test)	Overall MAE (Val)	Overall MAE (Test)
ElasticNet	0.575	0.597	90.723	88.652
RF	0.921	0.961	21.487	14.698
SVR	0.682	0.730	72.015	63.761
MLP	0.741	0.756	52.081	47.825
XGBoost	0.715	0.806	53.889	40.797

**FIGURE 6:**
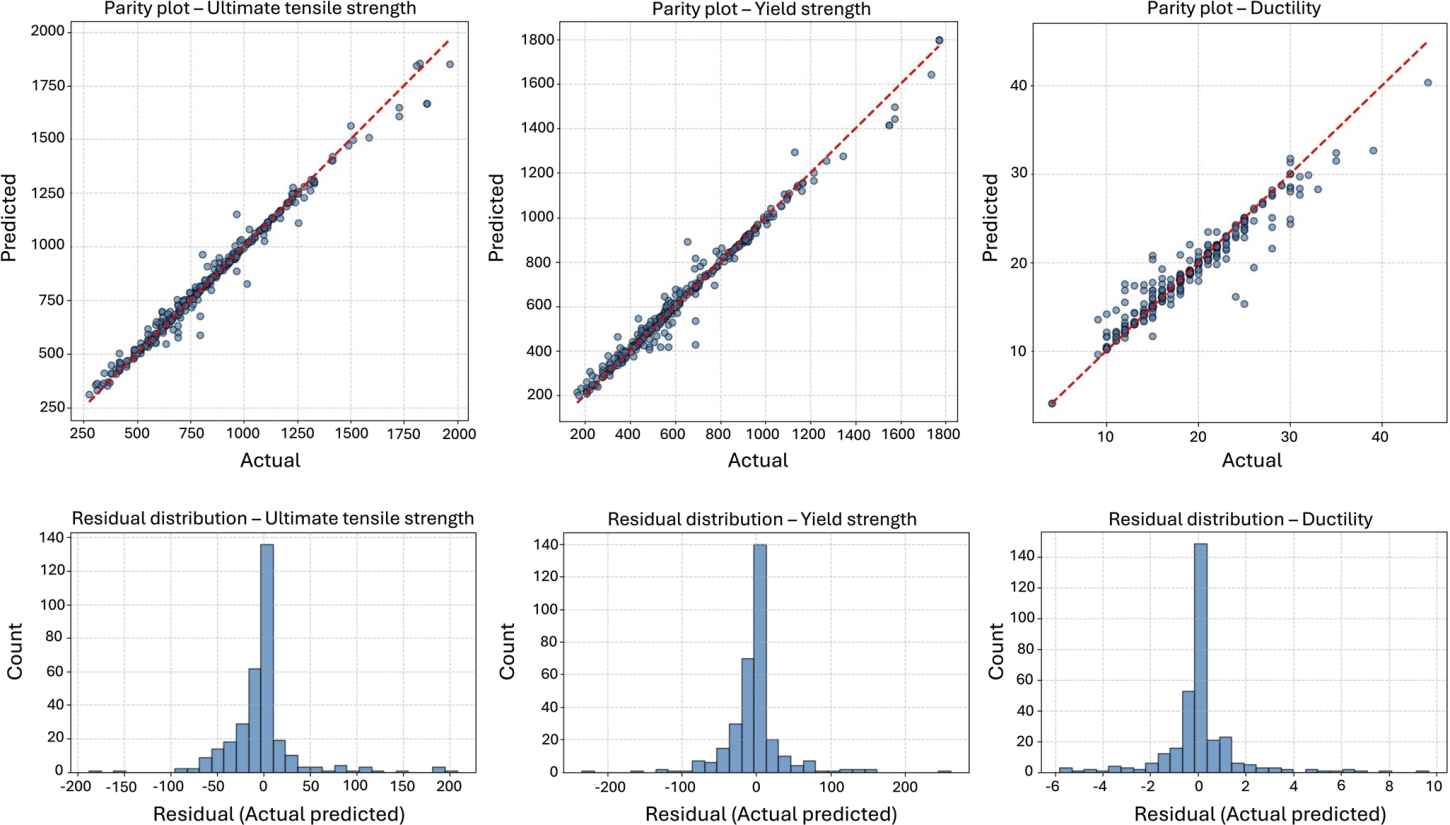
Parity plots and corresponding residual distributions for steel properties that arise from supervised ML using the Random Forest model. (left) ultimate tensile strength, (middle) yield strength, and (right) ductility.

As can be ascertained visually from Figure 5, of the models explored, the Random Forest (RF) model outperformed the other models. This is reinforced by the fuller range of performance parameters provided in Table 4. On this basis, the remainder of the study employed the RF model for the prediction of steel properties. To provide a more detailed expose of the performance of the RF model upon the steel database herein, Figure 6 has been prepared in order to reveal the parity plots and residual plots for each of ultimate tensile strength, yield strength and ductility when modelled with the RF. What is observed from Figure 5 is that predicted values align closely with actual data, and residuals cluster near 0, confirming a high accuracy and no bias.

## 4 Discussion

### 4.1 General Discussion

**FIGURE 7:**
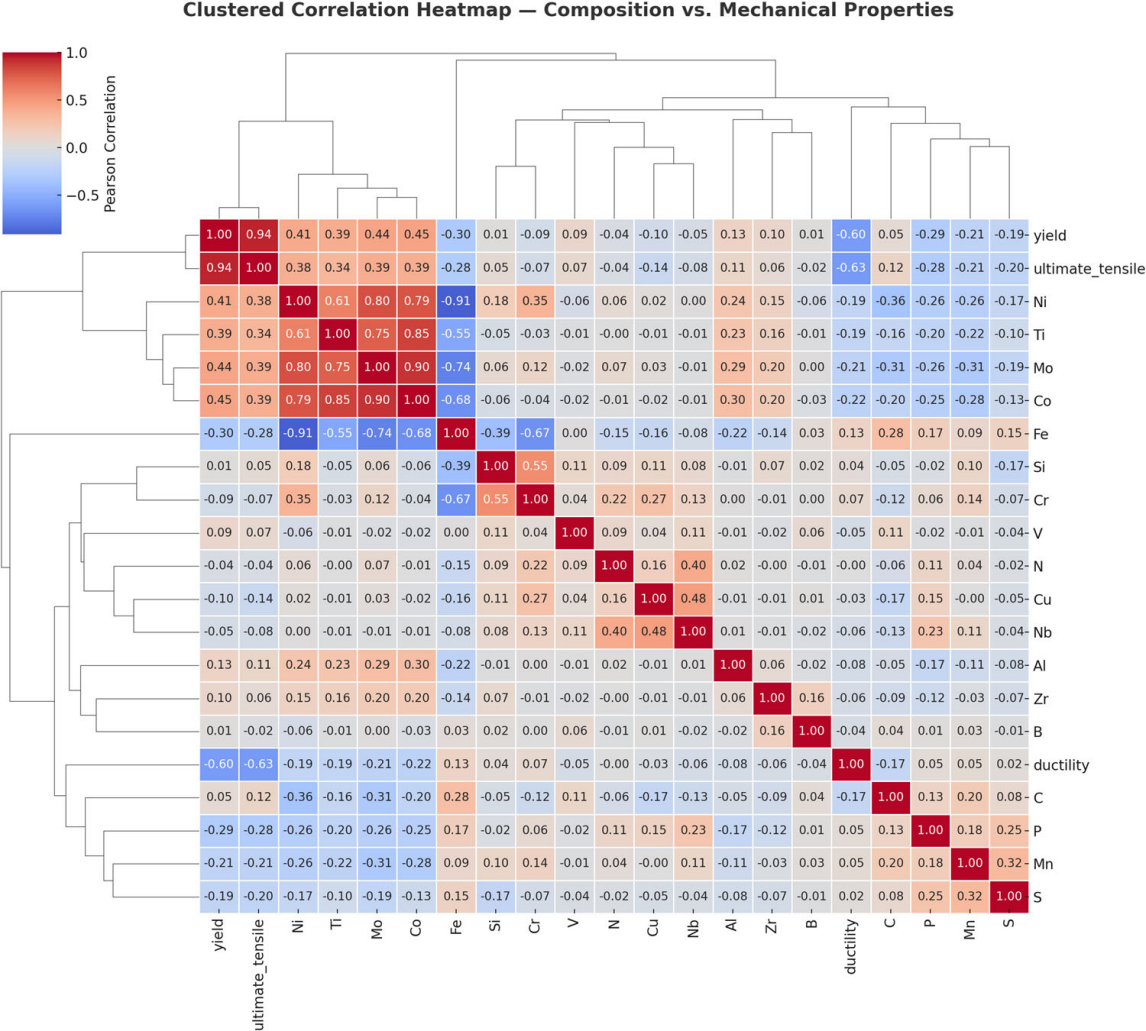
Clustered correlation heatmap of alloy composition and mechanical properties.

Whilst the unsupervised ML provided a detailed insight into the role of alloy processing on mechanical properties, to further assess the role of composition in the development of mechanical properties, a hierarchically clustered Pearson correlation matrix showing relationships between the principal alloying elements and mechanical properties is presented in Figure 7. Red and blue colours denote positive and negative correlations, respectively, with dendrograms indicating variable groupings based on correlation similarity.

From Figure 7, two dominant regions are evident: (i) a strength‐associated group comprising C, Cr, Mo, and Ni, reflecting their role in solid‐solution and precipitation strengthening; and (ii) a ductility‐associated group characterised by inverse correlations with the strengthening elements, capturing the intrinsic strength–ductility trade‐off. The resulting correlation structure delineates compositional families governing mechanical response in the studied alloys. Some additional extractions from the hierarchically clustered Pearson correlation matrix include the following points – with the caveat that only compositional effects are explored in Figure 7. Many such points are known from the literature [39], mechanistically; however, such abstractions from a data‐driven approach are important revelations on the basis of the interpretability of trends in a large dataset. The correlation hierarchy separates composition‐driven strength vs. ductility‐governed behavior (in the absence of processing). The dendrogram divides the dataset into two conceptual branches: One dominated by C, Cr, Mo, Mn, and Ni, correlating positively with strength; another with ductility, associating more closely with elements or clusters that do not increase strength (or even oppose it).

- Carbon (C) shows a dominant positive correlation with strength metrics

○ Both yield and ultimate tensile strength increase with carbon content (red block in the heatmap).
○ This aligns with well‐established strengthening mechanisms whereby carbon promotes solid solution strengthening and martensitic transformation hardening, particularly under quenched and tempered processing.
○ It is noted that the correlation of C with ductility is negative—highlighting the strength‐ductility trade‐off central to metallic alloys.

- Chromium (Cr), Molybdenum (Mo), and Nickel (Ni) tend to cluster together – forming a ‘hardenability triad’.

○ These elements often appear as a correlated sub‐cluster in the matrix, with modest positive correlation to strength.
○ Their synergy enhances hardenability and secondary carbide formation, contributing to higher yield and ultimate tensile strength after heat treatment.
○ Ni, while increasing toughness, still tracks positively with strength in this dataset—indicating it is part of a carefully balanced steel design.

- Manganese (Mn) and Silicon (Si) form a secondary strengthening correlation group.

○ Moderate positive correlation with yield and ultimate tensile strength.
○ Mn improves deoxidation and hardenability, while Si assists solid‐solution strengthening and contributes to ferrite stability.
○ Their co‐clustering reflects their similar role in ferritic–pearlitic steels and low‐alloy structural compositions.

- Phosphorus (P) and Sulfur (S) exhibit low or negative correlations with mechanical performance.

○ Weak or negative correlations with both strength and ductility reflect their impurity‐like behavior—promoting embrittlement and segregation.
○ This supports metallurgical expectations: both P and S are minimized in engineering steels for mechanical reliability.

**FIGURE 8:**
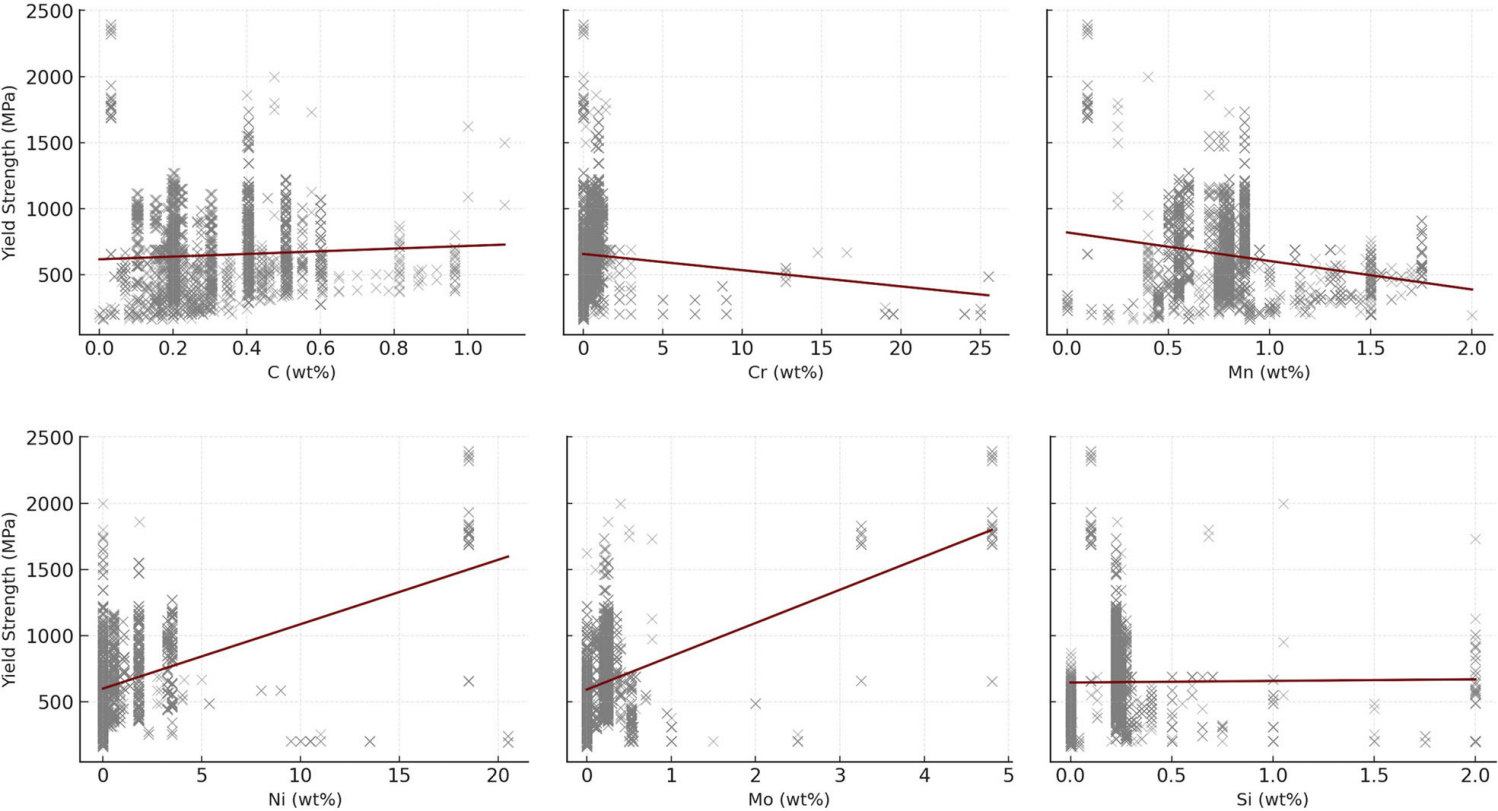
Yield strength versus concentration of C, Cr, Mn, Ni, Mo and Si, in steels in the database utilized in this study. The red line is a linear fit to the data.

In order to visualize the individual effects of alloying elements (presented independently, even in cases where forming part of a higher order alloy), Figure 8 presents yield strength as a function of C, Cr, Mn, Ni, Mo and Si content. What may be observed from Figure 8, is that whilst there are some ‘trends’ that track with composition (as depicted by poor linear fits in red), there is significant scatter in yield strength across a given elemental concentration. This scatter is not random noise, but is primarily attributable to the diverse processing conditions (i.e.,, the clusters detailed in Table 2 and Figure 2) within the dataset. This visually confirms the necessity of accurately clustering processing history before attempting property prediction.

As typified by Figures 4 and 7, the comparative analysis between processing‐defined clusters and alloy composition reveals that processing exerts the primary control over mechanical performance. When data are grouped by processing condition, yield and ultimate tensile strengths segregate into distinct regimes: *quenched‐and‐tempered steels* dominate the high‐strength range, *normalized steels* occupy intermediate values, and *annealed or as‐rolled materials* form the ductile baseline. This stratification (Figure 4) is sharper and less scattered than any trend explained by individual compositional variables alone (Figures 7 and 8), underscoring the decisive influence of thermal history (and hence steel microstructure, albeit that microstructure itself was not explicitly explored herein).

Nevertheless, composition defines the achievable limits within each processing regime. C content remains the strongest compositional predictor of strength, closely followed by Cr, Mo, and Ni, which together enhance hardenability and temper resistance. Mn and Si contribute moderate solid‐solution strengthening and stability effects, while P and S remain detrimental to mechanical integrity. These relationships are consistent across clusters, suggesting that while processing sets the microstructural framework, chemistry fine‐tunes the response within it. It is noted that the processing clusters presented here represent one pragmatic and reproducible encoding of complex free‑text process histories; they are not intended to exhaust all possible representations, and indeed over‑encoding (i.e.,, treating every text description as unique) eliminates any learnable structure. The cluster‑based approach, therefore, reflects an explicit trade‑off between fidelity and tractability, consistent with the scope of the present work.

Given the above complexities in human‐level interpretation across diverse compositions and processing conditions, a supervised ML approach for rationalising steel properties is an important tool for both alloy rationalisation and future design. Such a paradigm is best implemented by a user‐interactive tool, which allows general users (and metallurgists) the opportunity to interact with the supervised ML model developed in this work, as elaborated below. While equilibrium CALPHAD and existing semi‐empirical tempering models were considered, their known limitations in describing metastable martensitic states, quench‐rate‐dependent transformations, and broader alloy chemistries [40, 41, 42, 43] preclude a physically meaningful one‐to‐one comparison in the present work.

### 4.2 User Tool

**FIGURE 9:**
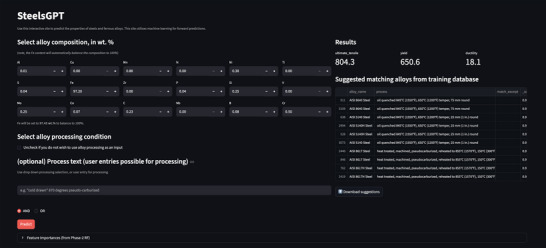
Screenshot of SteelsGPT, a user‐interactive site at https://steelsgpt.streamlit.app.

The Streamlit driven SteelsGPT website [38] allows users to input alloy compositions and select processing parameters to receive near instant predictions for key mechanical properties, including yield strength, ultimate tensile strength and ductility—along with alloy recommendations derived from the model outputs. This allows users to compare alloys, helping support informed material selection. SteelsGPT translates complex computational models into an accessible and interactive format that can be used directly by engineers, researchers, or students. Moreover, SteelsGPT predicts the properties of previously unexplored (novel) steel compositions. An image of the interface of SteelsGPT is provided in Figure 9.

### 4.3 Future Prospects

It is noted that the present work treats processing as a singular feature (albeit that this is an advance in the field of steels). The present work does not structurally encode the thermal–mechanical history, and therefore limits the model's extrapolation to novel conditions. Works such as those by Peng et al. [44] illustrate merit in future work, which may harness physics‐coupled machine learning, where synthetic microstructural or phase‐transformation features are embedded to improve both the model accuracy and interpretability (e.g. for Cr‐containing steels). The present study does not include microstructural features, with the development of properties implied by composition and processing method. A promising path forward is the development of microstructure‐aware data fusion, where compositional, processing, and microstructural information are integrated within a single predictive framework. Incorporating quantitative descriptors such as grain size, phase fractions, dislocation density, and carbide or precipitate morphology would allow models to capture the mechanistic origins of strength and ductility rather than relying solely on empirical correlations. Such integration can differentiate martensitic and bainitic transformations within nominally similar heat treatments, resolving the microstructural pathways that conventional data inputs cannot distinguish. Coupling high‐throughput microstructural characterisation with modern representation learning will advance this framework from data‐informed to physically interpretable alloy design—linking chemistry, processing, and structure to performance in a unified, predictive manner. Ultimately, this framework provides the foundation for the next logical step: moving from these predictive models to generative (inverse design) models. Future work will focus on leveraging generative AI to query the established property‐process‐composition space to answer the true design question: ‘Given a target set of properties, what are the most promising novel compositions and processing routes to achieve them?’. The best answer to that design question can, and should, include compositional complexity that also incorporates waste / recycled feedstocks [38] (which in turn, necessitates an ongoing contribution/expansion to the steel database presented herein, which is welcomed and essential for the progress of AI approached in sustainable steel design) [45].

## 5 Conclusions

- The analysis of clustering of textual processing descriptions for steels via NLP revealed that thermal history governs mechanical performance more decisively than composition alone. Quenched and tempered steels consistently occupied high‐strength regimes, while annealed and normalized clusters defined ductile baselines—confirming the dominant role of process‐induced microstructure.
- Alloy chemistry (i.e., composition) defines the performance envelope within each processing regime. Elements such as C, Cr, Mo, and Ni form a strength‐enhancing cluster, while P and S correlate negatively with both strength and ductility, reinforcing their role as embrittling agents (and reinforcing classical metallurgical principles through data‐driven insights.). These trends were extracted from high‐dimensional data using correlation matrices and cluster‐wise analysis.
- The framework presented herein couples unsupervised clustering with supervised machine learning (ML) to predict mechanical properties from composition and process descriptors. Among the tested ML models, the Random Forest model consistently outperformed others in accuracy and generalisability, achieving R^2^ >0.85 and MAE <15 MPa. This validates ensemble learning as a robust approach for metallurgical property prediction across heterogeneous datasets.
- A Streamlit‐based interface was developed to translate the ML pipeline into an accessible design tool (or, at the very least, a user exploration tool). Users may input alloy compositions and select processing routes to receive real‐time predictions of ultimate tensile strength, yield strength, and ductility. This interface bridges computational materials science with practical alloy selection and design.
- While the current models (and workflow) presented treat processing as clustered text embeddings and quantitate inputs as compositional entries, any future iterations should incorporate microstructural descriptors such as grain size, phase fractions, and precipitate morphology. Embedding physics‐aware features and coupling with generative models will enable extrapolation to novel steel chemistries and processing routes, advancing predictive alloy design beyond empirical boundaries.

## Conflicts of Interest

The authors declare no conflicts of interest

## Data Availability

The data that support the findings of this study are openly available in Mendeley at https://doi.org/10.17632/jmwb9ddd43.1, reference number [25].
